# Impacts of Drought and Rehydration Cycles on Isoprene Emissions in *Populus nigra* Seedlings

**DOI:** 10.3390/ijerph192114528

**Published:** 2022-11-05

**Authors:** Zhiyu Han, Yisheng Zhang, Houyong Zhang, Xuan Ge, Dasa Gu, Xiaohuan Liu, Jianhui Bai, Zizhen Ma, Yan Tan, Feng Zhu, Shiyong Xia, Jinhua Du, Yuran Tan, Xiao Shu, Jingchao Tang, Yingjie Sun

**Affiliations:** 1 School of Environmental and Municipal Engineering, Qingdao University of Technology, Qingdao 266520, China; 2 Guangdong-Hongkong-Macau Joint Laboratory of Collaborative Innovation for Environmental Quality, Guangzhou 511486, China; 3 Jinan Ecology and Environment Monitoring Center of Shandong Province, Jinan 250101, China; 4 Division of Environment and Sustainability, The Hong Kong University of Science and Technology, Hong Kong 999077, China; 5 Key Lab of Marine Environment and Ecology, Ministry of Education, Ocean University of China, Qingdao 266100, China; 6 LAGEO, Institute of Atmospheric Physics, Chinese Academy of Sciences, Beijing 100029, China; 7 Hebei Key Laboratory of Soil Ecology, Key Laboratory of Agricultural Water Resources, Center for Agricultural Resources Research, Institute of Genetic and Developmental Biology, Chinese Academy of Sciences, Shijiazhuang 050022, China; 8 School of Environment and Energy, Peking University, Shenzhen 518055, China

**Keywords:** drought, rehydration, isoprene, *Populus nigra*

## Abstract

The volatile organic compounds emitted by plants significantly impact the atmospheric environment. The impacts of drought stress on the biogenic volatile organic compound (BVOC) emissions of plants are still under debate. In this study, the effects of two drought–rehydration cycle groups with different durations on isoprene emissions from *Populus nigra* (black poplar) seedlings were studied. The *P. nigra* seedlings were placed in a chamber that controlled the soil water content, radiation, and temperature. The daily emissions of isoprene and physiological parameters were measured. The emission rates of isoprene (*F_iso_*) reached the maximum on the third day (D3), increasing by 58.0% and 64.2% compared with the controlled groups, respectively, and then *F_iso_* significantly decreased. Photosynthesis decreased by 34.2% and 21.6% in D3 in the first and second groups, respectively. After rehydration, *F_iso_* and photosynthesis recovered fully in two groups. However, *F_iso_* showed distinct inconsistencies in two groups, and the recovery rates of *F_iso_* in the second drought group were slower than the recovery rates of *F_iso_* in the first groups. The response of BVOC emissions during the drought-rehydration cycle was classified into three phases, including stimulated, inhibited, and restored after rehydration. The emission pattern of isoprene indicated that isoprene played an important role in the response of plants to drought stress. A drought–rehydration model was constructed, which indicated the regularity of BVOC emissions in the drought–rehydration cycle. BVOC emissions were extremely sensitive to drought, especially during droughts of short duration. Parameters in computational models related to BVOC emissions of plants under drought stress should be continuously improved.

## 1. Introduction

A considerable amount and types of biogenic volatile organic compounds (BVOCs) are exchanged between the vegetation and the surrounding air [1,2]. The global BVOC emissions reached 1150Tg in 1990, accounting for 90% of the global annual discharge of VOCs [3]. It plays a significant role in tropospheric chemical reactions, ozone generation, as precursors for secondary organic aerosols, global carbon budget, and new particles formation, with important feedbacks for air quality [4,5]. Isoprene is the key BVOC species considered in regional and global inventories since they are taken as representatives of the most important reactive portion of total emissions released by vegetation [6,7]. Isoprene plays a key role in tropospheric chemistry, the carbon budget, and global climate change, contributing to the formation of ozone and secondary organic aerosols in the atmosphere [8,9,10]. Consequently, changes in the emission of isoprene will affect atmospheric chemistry and ambient air quality on regional and global scales [11,12].

Drought is one of the most important natural disasters in the world and is known to severely impact ecosystem function [13,14]. Recent studies revealed increasing risks of prolonged drought periods and more frequent drought events due to changed precipitation patterns and rising temperatures [15]. Drought affects the physiological processes and growth of plants, such as net photosynthesis rates (*A*_net_), stomatal conductance (*g*_s_) and transpiration rate (*E*), that significantly correlate with BVOC generation [16].

Isoprene emissions are influenced by environmental factors such as temperature, solar radiation, plant water stress, and ambient ozone and CO_2_ concentrations [17,18,19,20]. The sensitivity of isoprene emissions to several environmental factors (e.g., temperature and radiation) has been well documented [21,22]. Drought is considered the key uncertainty factor in existing BVOC flux responses to global change processes [15]. It is of high importance to comprehend the BVOC emissions patterns of plants and ecosystems under drought stress, as uncertainty strongly limits the reliability of BVOC emission inventory models [23,24,25,26].

The response of isoprene emissions under drought stress is still very uncertain. Under drought stress in the early period, the emission rates of BVOCs will remain flat or increase to maximum values [27,28,29,30,31]. Significant reductions in isoprene emissions during severe and extreme droughts at the genetic, leaf, canopy, and ecosystem levels have been well described [27,32,33,34]. These studies suggest that the duration of stress appears to be the key predictor for emissions, as short-term stress increases emissions, while long durations of drought stress strongly suppress emissions [35]. The uncertainty of the isoprene emission inventory reached up to 40% [36]. In addition, Fortunati et al. [37] found that isoprene emission rates (*F*_iso_) of *Populus nigra* (black poplar) were not temperature-dependent during and after severe drought stress. Therefore, drought may surpass temperature and radiation and become the most important environmental factor affecting isoprene emissions [38]. However, understanding of the impact on the emission of isoprene under drought conditions is in its infancy, and even less is known about how the emissions of these different plants respond to two drought–rehydration cycle groups.

Drought disaster accounts for 50% of global meteorological disasters and is one of the most important natural disasters in the world [14]. Drought significantly affects global economic and ecosystem functions, reducing crop yields and affecting plant and animal growth development [39,40]. Arunrat et al., [39] studied how repeated drought in the future will impact crop yield; Skendzic et al. [40] confirmed that drought affected insect population dynamics. Drought is one of the most important factors in the process of plant growth, and impacts physiological metabolisms such as photosynthesis and stomatal conductance [41]. The highest emissions of isoprene normally occur in summer, which is the season with the most precipitation, alternating drought and rehydration [10,42]. The increase in temperature aggravates the occurrence of drought [38,43,44]. Different drought durations affect the ability of plants to adapt to different soil water content conditions, and plants may have to adjust their carbon allocation [45]. After plants experience variation in drought durations and precipitation, their effect on the isoprene emission rate needs to be explored. However, studies on isoprene emissions after rehydration under drought stress are limited, especially for rehydration after stress of varied drought durations.

The poplar plantation area exceeds 7 million hectares in China, ranking first in the world [46]. Isoprene is estimated to be about 650 Tg, mainly originating from deciduous trees [7]; poplar is the main species. Isoprene emitted by poplar is the precursor of ozone formation and plays a significant role in tropospheric chemical reactions [47]. Poplar is one of the most important models for studying isoprene emission, especially under stress [48]. *P. nigra* is therefore meaningful for the study of isoprene emissions under different drought or rehydration conditions. *P. nigra* is one of the most widely distributed species in China and has been selected and planted in many arid and barren places, such as the Three North Shelterbelt [49,50]. *P. nigra* is therefore meaningful for the study of isoprene emissions under different drought or rehydration conditions. The daily isoprene emissions from *P. nigra* under drought and rehydration treatment in a chamber were measured. The objectives of this study were: (1) to investigate the impact of short drought durations (~10 days) and long drought durations (~20 days) drought stress on the emissions of isoprene; (2) to explore the relationships between isoprene emission rates and photosynthetic parameters; (3) to compare the difference in isoprene emission rates under rehydration after different durations of drought; and (4) to hypothesize a theory to explain the mechanism of BVOC emissions in drought rehydration cycles of different durations.

## 2. Materials and Methods

### 2.1. Plant Material and Chamber Design

In September 2020, a total of 8 3-year-old *P. nigra* seedlings were obtained from a nursery in Qingdao, Northern China. The trees were transplanted to 50-L plastic pots (diameter 40 cm, height 50 cm) that contained 40% commercial potting soil and 60% clay soil. The soil mixture allowed fast drought application. These 8 pots were placed in a chamber without sun radiation, and the chamber size was 2.5 m × 3.5 m × 4 m (length × width × height). To provide consistent radiation, five 180 W light emitting diode (LED) lights (WEN-180, Guixiang Inc., Weifang, China) were installed. The LED lights were turned on at 7:00 a.m. and turned off at 6:00 p.m. during the whole experiment. The air circulation was controlled through an air intake and outlet pump, with a flow rate of 210 m^3^/h. The chamber temperature was maintained at 25 °C by an air conditioner. The chamber temperature and humidity were both recorded every minute (Supplementary Material Figure S1).

### 2.2. Drought Stress Experiments

#### 2.2.1. Soil Water Content

Soil water content (SWC) was monitored by a soil moisture sensor (EC-5, METER Group, Inc., Pullman, WA, USA), which was placed under 10 cm of soil. SWC was recorded every 30 s by a data collector (ZL6, METER Group, Inc., Pullman, WA, USA). To explore the wilting point of *P. nigra* in pots, a drought pre-experiment was set up (Supplementary Material Figure S2). Four trees were randomly selected without watering until the leaves and trees were completely wilted, and the duration was recorded. On D17, one piece of leaf was completely wilted, and on D22~D25, the whole trees were completely wilted. Therefore, the drought of the first group lasted 8~9 days, and the drought of the second group lasted 17 days. Two drought–rehydration cycle groups were performed in two independent experiments, each consisting of 2 replicates. In the first drought-rehydration cycle group (short drought durations), there were two drought-rehydration cycles. The first lasted for 8 days, and 2 L of water was rehydrated on the night of D8. The second drought stress lasted 9 days, and 2 L of water was rehydrated on the night of D18. In the second drought-rehydration cycle group (long drought durations), the drought-rehydration cycle lasted for 17 days, and 2 L of water was rehydrated on the night of D19.

#### 2.2.2. Isoprene Sampling and Gas Exchange

The emissions of isoprene were sampled by a portable photosynthetic apparatus (CIRAS-3, PP Systems Inc., Hitchin, UK) in the chamber. Fresh air was filtered into the leaf chamber (18 mm × 25 mm) of a portable photosynthetic apparatus with a flow rate of 100 mL/min. The sampling temperature and photosynthetic active radiation (PAR) in the leaf chamber were 30 °C and 1000 µmol m^−2^ s^−1^, respectively. The sample was pumped into the adsorption tube (Markes International Ltd., UK) through the pump (GilAir plus, Sensidyne Gilian Inc., Petersburg, FL, USA). The flow rate was set at 90 mL/min and lasted for 30 min. Net photosynthetic rate (*A*_net_), intracellular carbon dioxide concentration (*C*_i_), stomatal conductance (*G*_s_), transpiration rate (*E*), water use efficiency (*WUE*), and vapor pressure deficit (*VPD*) measurements were performed using the portable photosynthetic apparatus CIRAS-3.

#### 2.2.3. Quantification of Isoprene

The gas chromatography/mass spectrometry (GC/MS) system used an Agilent 5977B GC/MSD coupled to an Agilent 7890B GC. The desorber was UNIT-xr (Markes International Ltd., Bridgend, UK) with an automatic sample processor (ULTRA-xr, Markes International Ltd., Bridgend, UK). The initial oven temperature was increased at 20 °C min^−1^ from 40 to 280 °C. The desorbed isoprenoids were cryofocused at −3 °C for 2 min, after which the cryotrap was heated rapidly to 280 °C and placed into a 30 m×0.32 mm × 0.18 μm column (DB-624, Agilent (J&W), Santa Clara, CA, USA). The flow of helium was 1 mL/min, and the total run time was 38 min, including a solvent delay of approximately 2 min. The initial oven temperature was increased on the capillary column at 5 °C min^−1^ to 200 °C, then increased with a 20 °C min^−1^ ramp to 260 °C and maintained at 260 °C for 2 min.

### 2.3. Quality Assurance/Quality Control

All *P. nigra* seedlings were exposed to the same temperature and radiation. The isoprene emission and photosynthetic parameters on D1 were used as controls. There were two trees in each of the two groups. One leaf was selected from each tree that had a similar size, degree of development and height. Increases or decreases in isoprene emissions and photosynthesis during drought and rehydration were calculated as the treatment effect, which equaled the treatment minus the control and was divided by the control. A pair comparison test (mean comparison) was applied to compare isoprene emissions over the drought rehydration cycle (from D2 to D24) with those from D1 (control). All statistical tests were considered significant at *p* < 0.05. Error bars represent the standard deviation of the two independent experiments.

## 3. Results and Discussion

### 3.1. SWC and Physiological Parameters

**Figure 1:**
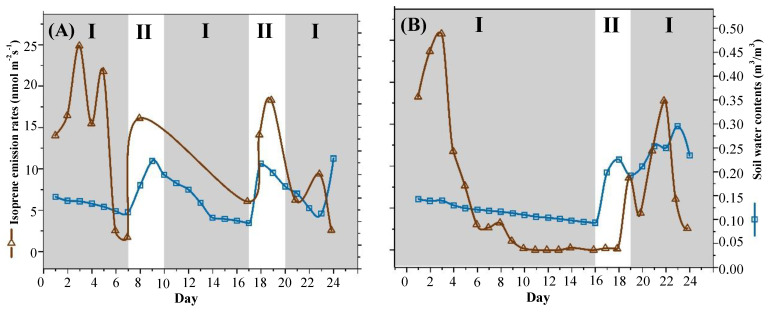
SWC and isoprene emission rates in drought–rehydration cycles of different drought durations. (**A**). SWC and isoprene emission rates in a drought-rehydration cycle of short drought duration. (**B**). SWC and isoprene emission rates in a drought-rehydration cycle of long drought duration. ‘I’ represents the drought period; “II” represents the rehydration period. (Note, The *F_iso_* data of short drought durations in D9-D15, and D20 was lost).

The *F*_iso_ and SWC are shown in Figure 1. The SWC of the first group was 0.152 ± 0.002 m^3^/m^3^ on D1. With increasing drought stress, the SWC value decreased to 0.117 ± 0.013 m^3^/m^3^ on D8, and the lowest was 0.096 ± 0.007 m^3^/m^3^ on D17 (Figure 1A). The SWC in the second group was 0.143 ± 0.002 m^3^/m^3^ on D1, and with the deepening of drought, the SWC gradually decreased to 0.093 ± 0.003 m^3^/m^3^ on D16 (Figure 1B).

**Figure 2:**
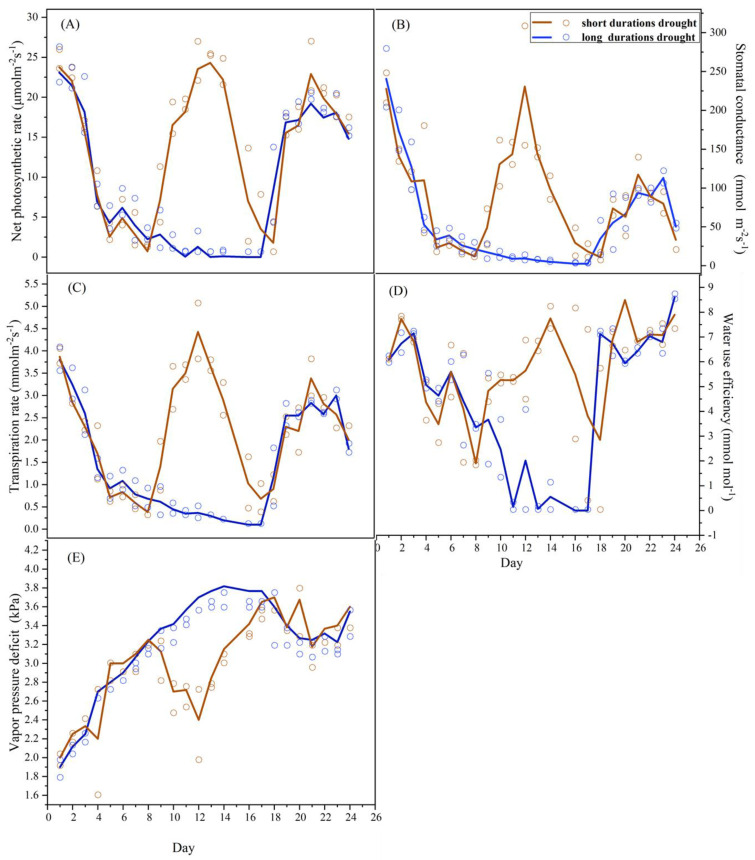
The changes in net photosynthetic rate, stomatal conductance, transpiration rate, water use efficiency, and vapor pressure deficit during short and long durations of drought rehydration cycles. (**A**). The changes in net photosynthetic rate in short and long durations of drought rehydration cycles. (**B**). The changes in stomatal conductance in short and long durations of drought rehydration cycles. (**C**). Changes in transpiration rate in short and long durations of drought rehydration cycles. (**D**). Changes in water use efficiency in short and long durations of drought rehydration cycles. (**E**). The changes in vapor pressure deficit in short and long durations of drought rehydration cycles.

The change in the gas exchange parameters is shown in Figure 2. Drought stress obviously limited the *A*_net_ (Figure 2A), *g*_s_ (Figure 2B), *E* (Figure 2C), and *WUE* (Figure 2D), which gradually decreased with the deepening of the drought, but the *VPD* (Figure 2E) showed the opposite trend in both groups. In the first and second groups, the decreasing trend and size of the five physiological parameters showed obvious consistency (Figure 2). The *A*_net_ and *g*_s_ dropped rapidly by more than 90% in the first five days of the drought. Drought stress is the largest limiting factor for poplar growth, especially for photosynthesis [16]. On the one hand, drought stress is the direct reduction of photosynthetic raw materials, and on the other hand, drought stress indirectly limits *g*_s_ and enzyme activity reduction [41,51].

### 3.2. Isoprene Emission Rates under Drought Stress

The *F*_iso_ of the control was 15.7 ± 2.3 nmol m^−2^s^−1^. In the first and second groups, *F*_iso_ had an initial stimulation followed by a dramatic decrease from D4 to D6 (Figure 1). There is a high consistency of *F*_iso_ between different drought durations, such as the time of changes in the peak of *F*_iso_. On D3, the maximum *F*_iso_ values in the first and second groups were 24.9 ± 1.05 nmol m^−2^s^−1^ and 25.8 ± 2.50 nmol m^−2^ s^−1^, respectively. On D4, *F*_iso_ rapidly decreased, and on D6, *F*_iso_ decreased to almost zero.

Isoprene emission was stimulated by drought stress, regardless of exposure to drought–rehydration cycles of different durations (Figure 1). On D3 in the first and second groups, compared with the control, *F*_iso_ increased by 58.0% and 64.2%, respectively. These results were consistent with the results obtained from studies on the effects of water stress on isoprene emission [27,52]. After 6 days of drought stress, the *F*_iso_ in *Alnus glutinosa* on D6 was higher than the *F*_iso_ in *Alnus glutinosa* on D1 [29]. Limited studies clearly identified that under drought stress, isoprene emissions were briefly stimulated, increasing by 33.7% to 300% (Table 1). Our results fall within the range, and that peak occurred after D3 in both groups. The stimulated *F*_iso_ in our study was relatively lower than previous results (33.7–300%). However, other studies showed either almost no change in isoprene emission or slightly lower than the control [31,37,53,54], possibly attributed to the low time resolution (not daily measurement) of BVOC emissions, which might miss the peaks of BVOC emissions. Pegoraro et al. [55] studied *Quercus virginiana* Mill, and *F*_iso_ remained essentially constant for 8 days of treatment. Whether this threshold is common to all species remains uncertain.

The rare isoprene emitter *Hakonechloa macra*, compared with the stronger isoprene emitter, was observed to impair chloroplast ultrastructure, indicating damage to photosynthetic machinery under drought conditions [54,60]. There are three possible explanations: (1) isoprene is an effective antioxidant; drought can promote oxidation by increasing the oxidative pressure of plant cells, and isoprene protects plant cells from oxidative damage [52,57]; (2) membrane stabilizers protect cells of plants such as thylakoids and chloroplasts during drought [60,61]; and (3) membrane stabilizers reduce the damage of reactive oxygen species (ROS) to plants and suppress the generation of ROS [62,63,64].

However, as SWC decreased, progressive and steady declines in *F*_iso_ under stress conditions were observed; on D7 in the first groups, the decline in *F*_iso_ decreased to 1.73 ± 0.018 nmol m^−2^s^−1^, and on D10 in the second drought, the decline in *F*_iso_ decreased to zero. The results were consistent with previous studies [31,34,65,66]. Beckett et al. [27] studied *Xerophyta humilis* subjected to severe drought treatment. When the relative soil water content (RWC) decreased to 80%, the *F*_iso_ peaked, and when the RWC decreased to 53%, the *F*_iso_ decreased by zero. The second group had a longer drought, and the plants needed more effective protection; isoprene is increased when desiccation is moderate, while nonvolatile isoprenoids operate when drought stress is more extreme [27,57].

### 3.3. Isoprene Emissions and Physiological Parameters during Rehydration

**Figure 3:**
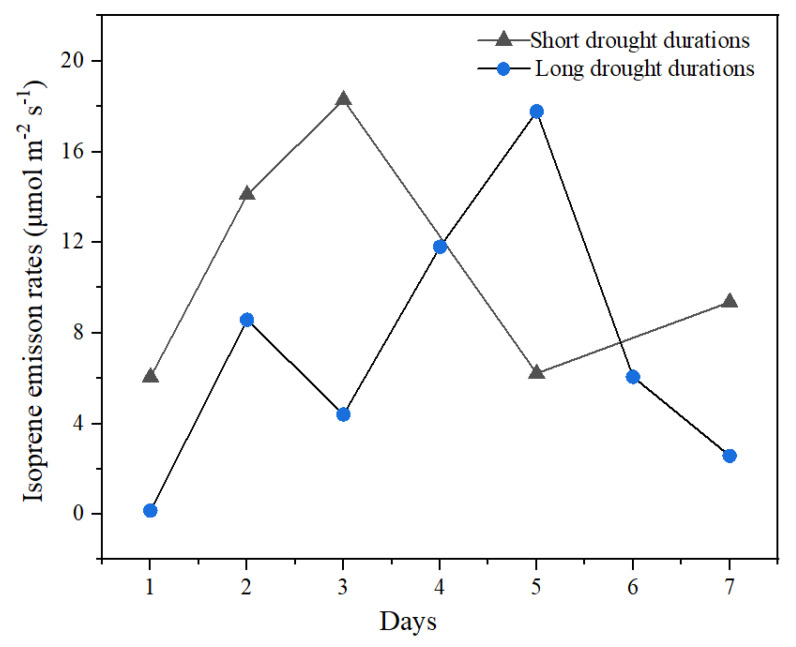
The isoprene emission rates during rehydration after short and long drought durations.

The difference in the isoprene emission rates of *P. nigra* between the first and second groups during rehydration is shown in Figure 3. After rehydration, *F*_iso_ in the first and second groups showed distinct inconsistencies (Figure 3), but physiological parameters showed clear consistency (Figure 2). After rehydration, emissions of isoprene recovered at a slower rate than photosynthesis. *F*_iso_ was slightly different in the two groups after rehydration. After rehydration in the first and second groups, the maximum *F*_iso_ was 18.3 ± 0.238 nmol m^−2^s^−1^ and 17.8 ± 0.475 nmol m^−2^s^−1^, respectively, which increased by almost 10% compared with the control.

Full recovery of *A*_net_ occurred after rehydration, confirming that photosynthetic limitations were fully reversible and that no permanent damage occurred. On D3 of rehydration, the *A_net_*, *E* and *WUE* of the first and second groups were fully recovered. However, after the second rehydration of the first group, the *g*_s_ and *VPD* recovered less than 50% compared to the control. After the first rehydration of the first groups, the *g*_s_ and *VPD* increased rapidly and almost recovered to the control level (Figure 2B,E). This result indicates a complete recovery of photosynthesis and no permanent limitations caused by drought. This pattern was in good agreement with the characteristic adaptation strategy of this species to withstand summer drought [67].

After rehydration, the *F*_iso_ completely recovered, which is in line with others reporting on isoprene emitters (Table 2), such as *Arundo donax, Populus alba* and *Quercus virginiana* Mill [53,55,60]. Limited studies have investigated the effects of rehydration after drought stress on isoprene emissions for *P. nigra*. Fortunati et al. [37] found that after a 3-day rehydration phase, the *F*_iso_ of *P. nigra* recovered in plants grown at both 25 and 35 °C. However, isoprene emissions did not reach the prestress levels even 15 days after rehydration; when photosynthesis had completely recovered, the reduction of isoprene emissions after recovering from drought stress was particularly strong in leaves grown at 35 °C. Previous studies of rehydration showed that isoprene emitters could still recover and increase within a short period even under severe drought conditions. Brilli et al. [53] found that after rehydration of *Populus alba* under severe drought treatment, *F*_iso_ was restored to 57%, 160% and 120% of the control on D2, D7 and D14, respectively. Upon full rehydration to 100% of soil water content, isoprene emission reached levels of 3.6–5.2 nmol m^−2^s^−1^, which was equivalent to the maximum emission rate upon dehydration and much higher than prior to dehydration in *X. humilis* [27], demonstrating that drought stress, even severe drought, did not affect *P. nigra* production or the emission of isoprene.

### 3.4. The Impact of Physiological Parameters on Isoprene

In the second group, *F*_iso_ and the physiological parameters were significantly positively correlated. Among these compounds, *g*_s_ had the highest correlation. Generally, the first event characterizing the plant response to water shortage is progressive stomatal closure triggered by increased stomatal closure [68], a significant effect of *F*_iso_ [59,69]. In the drought rehydration cycle, *A*_net_ and *g*_s,_ the main photosynthetic factors affecting isoprene emissions, have previously been reported [16,55,59]. However, a lower drought sensitivity of *F*_iso_ compared with *A*_net_ and *g*_s_ was found; for example, on D3, *F*_iso_ increased by 42.5% in the second group, but *A*_net_ and *g*_s_ decreased by 47.1% and 37.6%, respectively, consistent with other results demonstrating the response to drought stress [16,55,59]. Pegoraro et al. [55] studied *Quercus virginiana* Mill saplings. *A*_net_ and *g*_s_ decreased by 92% and 91%, respectively, while *F*_iso_ remained essentially constant for 8 days of treatment and for 12 days under severe drought conditions, and *F*_iso_ was reduced by 64%. Seco et al. [34] found that in the Ozark area where extreme drought occurred, the net flux of CO_2_ reached its seasonal maximum approximately a month earlier than isoprenoid fluxes, highlighting the different responses between isoprene emissions and physiological parameters to drought stress, and previous studies confirmed that the different responses progress under drought conditions [37,67]. This addition of isoprene emissions seemed to be relatively independent from photosynthesis, indicating a more complex regulation of isoprene emissions [31,57,60,69]. In these cases, an uncoupling between isoprene emissions (that remains stable or decreases slightly) and photosynthesis (that decreases dramatically) has been observed for different plant species [27,57].

Furthermore, as pointed out in our previous investigations, isoprene is important for plants to withstand drought stress. Plants produced high isoprene concentrations under environmental stress conditions because of a low allocation of carbon to growth, suggesting a trade-off between growth and defense [70]. Previous findings confirmed that isoprene emission was not inhibited by drought stress-induced stomatal closure and that isoprene emission was uncoupled from *A*_net_ under drought stress conditions [27,60]. The ability to use stored carbon (alternative carbon sources), as opposed to assimilated photosynthate, for isoprene production might be important as plants routinely experience photosynthetic depression in response to environmental stress [16,33,53,59]. Isoprene emission has been proposed to possibly be regulated by substrate availability. Studies using ^13^C isotopes in plants have confirmed that mainly carbon sources perform photosynthesis in well-watered plants, and the percentage contribution of these additional carbon sources greatly increases under stress conditions. In well-watered plants, 75~88% of the carbon in isoprene was derived from photosynthate. Under moderate stress and drought stress it dropped to 60%, but this percentage dropped significantly under severe stress to only 10~20% [16,53,59]. Thus, increasing the duration of drought led to an increased contribution of alternative carbon sources to the 2-C-Methyl-D-erythritol 4-phosphate (MEP) pathway, rather than newly made products of photosynthesis [60]. In the early stages of drought, isoprene was used as a protection against drought. As the duration of drought increased, *F*_iso_ experienced a very significant reduction, and nonvolatile isoprenoids in the MEP pathway, such as zeaxanthin and lutein, were found to be produced in large quantities using alternative carbon sources to cope with drought stress [27,33].

**Figure 4:**
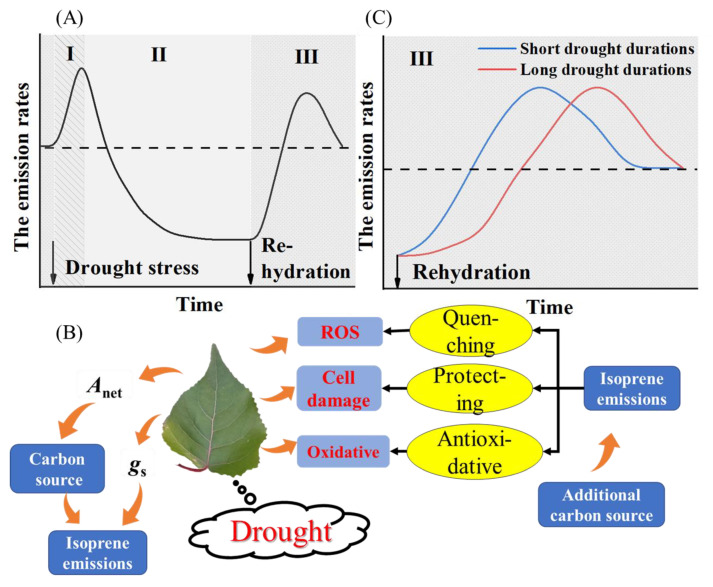
The response of BVOC emissions to the drought-rehydration cycle is divided into phases I, II and III. (**A**) shows the changes in BVOC emissions in phases I, II and III of the drought rewatering cycle. (**B**) shows the stimulation of BVOCs by drought stress in phase I. (**C**) shows the effect of short and long drought durations on the recovery rate of BVOCs in phase III.

Based on our results and a review of previous research on the leaf-level response of isoprene emissions to the drought-rehydration cycle, we hypothesize that the response has three phases (Figure 4). The drought-rehydration cycle is divided into phases I, II and III. Figure 4A shows the changes in BVOC emissions in phases I, II and III of the drought rewatering cycle. Figure 4B shows that in phase I, the stimulation of BVOCs by drought stress is the focal point of the debate. Figure 4C shows the effect of short and long drought durations on the recovery rate of BVOCs in phase III. In phase I of mild drought stress, emissions were stimulated using alternative carbon sources and used to protect plants against the stress, even though reduced values of stomatal conductance were associated with the physiological response to drought stress (Figure 4A,B). In phase II of more severe drought stress, emissions were suppressed by reductions in substrate availability and/or isoprene synthase transcription (Figure 4A). In the III phase of rehydration, emissions recovered fully (Figure 4A), but the recovery rate of emissions under long-duration drought conditions was slower than the recovery rate of emissions under short-duration drought conditions (Figure 4C). The hypothesis for phase I of Figure 4A is based on the observation that drought stress enhances BVOC emissions (Table 1). The second part of the hypothesis (Figure 4B) is based on numerous observations [16,52,53,57,59,60,61,62,63,64], while the last part of the hypothesis (Figure 4C) is based on observations and theoretical considerations [35]. 

## 4. Conclusions

*A*_net_, *g*_s_, *E*, and *WUE* gradually decreased with increasing drought, but *VPD* showed the opposite trend. The *F*_iso_ of the control was 15.7 ± 2.3 nmol m^−2^s^−1^. *F*_iso_ showed an initial stimulation followed by a dramatic decrease when the stress was severe. On the third day of the first and second groups, *F*_iso_ increased by 58.0% and 64.2% compared to the control, respectively. After rehydration, *F*_iso_ under drought stress showed distinct inconsistencies in the first and second groups. Isoprene emissions recovered at a slower rate than photosynthesis.

Further research is necessary to determine the change in isoprene emission rates in the drought rehydration cycle. As the world’s climate changes, such knowledge may be especially valuable for boreal tree species BVOC emission inventories, such as those in China.

Emission rates may be underestimated for isoprene, which show fast reactions with ozone. To further unravel the effect of long-term drought-rehydration on isoprene emissions, more studies characterizing emission patterns in predrought and rehydration periods are needed. These results highlight that direct plant stress sensing creates opportunities to understand the overall complexity of stress-related BVOC emissions.

## Figures and Tables

**Table 1:** Summary of increased rates on BVOCs emissions under drought stress.

Plant	Location	Emission Rates		Increased Rates (%)	Reference
		Pre-Stress	Stress		
*P. nigra*	Lab	15.7 nmol m^−2^s^−1^	24.9 nmol m^−2^s^−1^	58.0	Short durations (this study)
*P. nigra*	Lab	15.7 nmol m^−2^s^−1^	25.9 nmol m^−2^s^−1^	64.2	Long durations (this study)
*Quercus ilex*	Prades forest, Catalonia	N.A	N.A	68	[32]
*Xerophyta humilis*	Lab	1 nmol m^−2^s^−1^	4 nmol m^−2^s^−1^	300	[27]
*Pinus massoniana*	Lab	N.A	N.A	190	[56]
*Ficus septica*	Lab	N.A	N.A	160	[57]
*Cistus monspeliensis*	Natural Reserve, Italy	210 nmol m^−2^s^−1^	340 nmol m^−2^s^−1^	61.3	[58]
*Quercus pubescens*	A forest in France	78.4 μgC^−1^gDMh^−1^	104.8 μgC^−1^g DMh^−1^	33.7	[26]
*Populus deltoides*	Lab	37.6 nmol m^−2^s^−1^	48.8 nmol m^−2^s^−1^	37.4	[59]

**Table 2:** The isoprene emission rates in rehydration durations of this study and other studies.

Plant	Drought Durations	Rehydr-ation	Emission Rates (nmol m^−2^s^−1^)			References
			Rehydration	Pre-Stress	Stress	
*P. nigra*	8 Days	3 Days	18.3	18.3	18.3	Short durations (this study)
*P. nigra*	17 Days	5 Days	17.8	17.8	17.8	Long Durations (this study)
*Quercus virginiana* Mill.	12Days	4 Dys	20.5	20.5	20.5	[55]
*Robinia pseudoacacia* L.	N.A	N.A	Completely recover	Completely recover	Completely recover	[68]
*Xerophyta* *humilis*	RWC 0%	RWC 100%	5.2	5.2	5.2	[27]
*Populus alba*	FTSW5	7 Days	24.58	24.58	24.58	[53]

Notes: RWC is relative water content; FTSW5: fraction of transpirable soil water is 5%.

## Data Availability

Not applicable.

## Supplementary Materials

The following are available online at https://www.mdpi.com/article/10.3390/ijerph192114528/s1, Figure S1: Chamber design. Figure S2: Pre-experiment was set up, with *Populus nigra* leaf and seedling wilting. (A) In the long-term drought a leaf is completely wilted on D16. (B) After 22~23 days of drought, the *Populus nigra* seedling were completely wilted. Figure S3: Under short and long drought durations, the value for chlorophyll SPAD.
