# Supplementary material for: Impacts of Drought and Rehydration Cycles on Isoprene Emissions in Populus nigra Seedlings
Source: Int J Environ Res Public Health. 2022 Nov 5;19(21):14528. doi: 10.3390/ijerph192114528 (PMC9655116; doi:10.3390/ijerph192114528)
Supplement: Supplementary file 1 [file ijerph-19-14528-s001.zip › ijerph-1960375-supplementary.pdf]

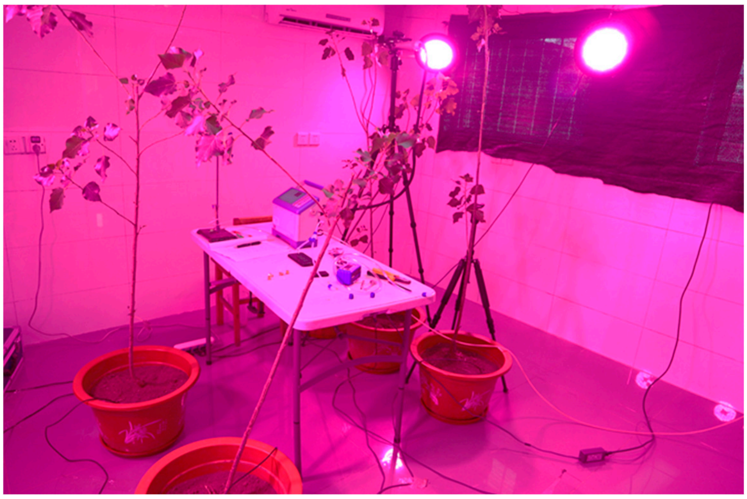

Figure S1. Chamber design.

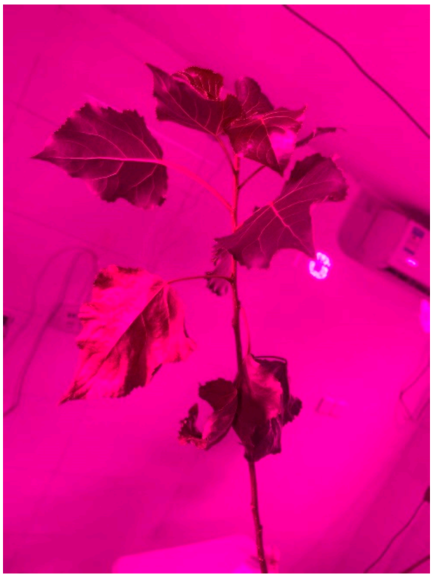

(**A**)

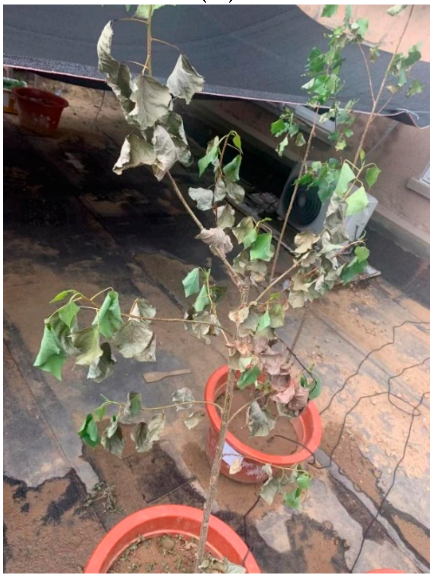

(**B**)

Figure S2. Pre- experiment was set up, which *Populus nigra* leaf and seedling wilting. (**A**)In the long-term drought a leaf is completely wilted on the D16. (**B**) After 22~23 days of drought, the *Populus nigra* seedling were completely wilted.

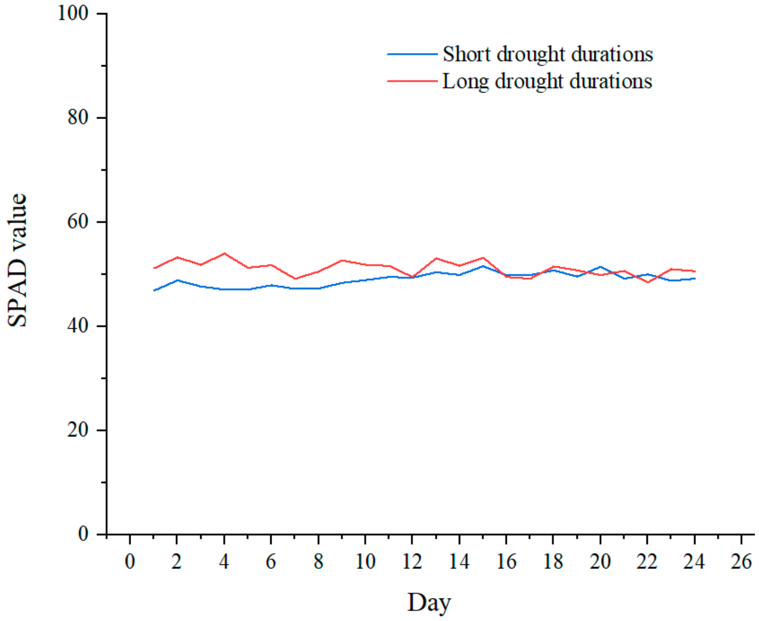

Figure S3. Under short and long drought durations, the value for chlorophyll SPAD.
